# Resting-state functional connectivity correlates of gait and turning performance in multiple sclerosis: a multivariate pattern analysis

**DOI:** 10.1038/s41598-025-21102-6

**Published:** 2025-10-20

**Authors:** Clayton W. Swanson, Anthony D. Gruber, Sutton B. Richmond, Torge Rempe, David J. Clark, Brett W. Fling

**Affiliations:** 1 School of Kinesiology, College of Health Science, Boise State University, Boise, ID USA; 2 Brain Rehabilitation Research Center, Malcom Randall VA Medical Center, Gainesville, FL USA; 3 Department of Neurology, College of Medicine, University of Florida, Gainesville, FL USA; 4 Department of Health and Exercise Science, Colorado State University, Fort Collins, CO USA; 5 Molecular, Cellular, and Integrative Neuroscience Program, Colorado State University, Fort Collins, CO USA

**Keywords:** Multiple sclerosis (MS), Resting-state functional connectivity, Multivariate pattern analysis (MVPA), Gait performance, Turning kinematics, Neural network connectivity, Multiple sclerosis, Multiple sclerosis

## Abstract

Multiple sclerosis (MS) often leads to mobility impairments, yet the neural mechanisms underlying these deficits remain poorly understood. This study examined whether resting-state functional connectivity (rs-FC) differs between people with MS (PwMS) and healthy controls in relation to spatiotemporal mobility performance. We hypothesized that group differences within the default mode (DMN), frontoparietal (FPN), somatomotor (SN), and visual (VIS) networks would be associated with gait and turning metrics. Twenty-nine PwMS and 28 matched controls completed a two-minute walk test, 180° walking turns, and 360° in-place turns at natural and fast speeds. fMRI data were analyzed using multivariate pattern analysis (MVPA) and post-hoc seed-to-voxel analyses for gait speed, cadence, double support time, stride length, turn duration, peak velocity, and turn angle. PwMS exhibited slower gait speed, shorter stride length, and impaired 360° turning, but no group differences in cadence, double support, or 180° turn metrics. MVPA revealed rs-FC differences across DMN, FPN, SN, and VIS networks. While rs-FC differences were evident for walking metrics, within-group associations were not significant. In contrast, 360° turn angle showed distinct within-group rs-FC associations, particularly involving VAN and DAN networks. These findings highlight turning as a sensitive task for capturing functional neural differences in MS.

**Supplementary Information:**

The online version contains supplementary material available at 10.1038/s41598-025-21102-6.

## Introduction

Multiple sclerosis (MS) is a chronic neurodegenerative disease characterized by CNS demyelination, often leading to progressive mobility impairments, including deficits in walking and turning^1–5^. Mobility dysfunction is therefore a primary concern for three-quarters of people with MS (PwMS), significantly impacting their quality of life^6^. Given its strong link to CNS damage, mobility impairments in MS have been increasingly studied using structural and functional neuroimaging, revealing significant associations with neural changes^5,7–11^.

Functional magnetic resonance imaging (fMRI) enables the study of intrinsic functional connectivity, which reflects the coherence of neural activity between brain regions at rest or during tasks. Resting-state fMRI (rs-fMRI) assesses intrinsic networks by analyzing spontaneous low-frequency fluctuations in the blood-oxygen-level-dependent (BOLD) signal, revealing connectivity patterns across anatomically distinct regions^12,13^. Many of these networks are disrupted in MS, with alterations linked to disease severity and behavioral deficits, including motor dysfunction. While task-based fMRI has traditionally been used to study connectivity in MS, recent research has increasingly focused on rs-fMRI, particularly within the default mode network (DMN)^13^. Studies have reported associations between altered connectivity and disability measures, cognitive impairment, and fatigue^14–16^. Locomotor performance has also been associated with functional connectivity in motor and non-motor networks (e.g., DMN)^11,17^. However, little research has examined whole-brain connectivity in relation to detailed kinematic measures of linear and non-linear walking.

Conventional rs-fMRI analyses often focus on a-priori selected brain regions or networks, limiting the scope of connectivity assessment. While this approach maintains statistical power, it may overlook broader functional connectivity patterns beyond predefined regions of interest (ROIs)^18^. In contrast, data-driven methods such as multivariate pattern analysis (MVPA) allow whole-brain connectivity analysis at the voxel level, offering a more comprehensive investigation of functional connectivity dynamics^18^. MVPA can identify functional connectivity patterns associated with behavioral measures, which can then be further examined using conventional seed-to-voxel methods^19^. To our knowledge, this is the first study to use MVPA to investigate rs-fMRI connectivity differences related to walking and turning performance in PwMS and healthy controls.

This study aimed to characterize how functional connectivity patterns differ between PwMS and healthy controls in relation to spatiotemporal gait and turning metrics using an MVPA-based approach. We hypothesized that linear and non-linear gait metrics would be associated with rs-fMRI connectivity patterns in frontal, parietal, occipital, and temporal regions, particularly within the DMN, frontoparietal network (FPN), somatomotor network (SN), and visual network (VIS) as defined by the Yeo et al. (2011) atlas^17,20–22^. Additionally, we expected that PwMS and healthy controls would exhibit distinct associations between functional connectivity and mobility performance.

## Methods and materials

### Population

Twenty-nine adults with relapsing-remitting multiple sclerosis (RRMS) and 28 healthy controls participated in the cross-sectional study, each completing two laboratory visits. Healthy controls were recruited to match the MS group in age and sex at the group level, minimizing potential confounding effects of these demographic variables on between-group comparisons. Participants were excluded if they: could not walk or stand for 10 min without an assistive device, had MRI contraindications, or had musculoskeletal or vestibular conditions. Healthy controls were also required to be free from clinically diagnosed neurological or mobility-affecting conditions. Further participant details are provided in Table 1. The study was approved by the Colorado State University Institutional Review Board (IRB#: 18–7738 H), all participants provided written informed consent, and all experiments were performed in accordance with relevant guidelines and regulations.

### Walking and turning collection

Walking performance was assessed using a self-paced two-minute walk test (2MWT) along a 27-meter hallway. Turning performance was evaluated through three separate 360° turn trials at a self-selected fast pace, one minute of continuous alternating 360° turns at a natural pace, and 180° turns during the 2MWT. Before each trial, foot placement was standardized using a template, and participants were instructed to stand still with a forward gaze. During the 2MWT and 180° turns, participants were asked to turn as if retrieving a forgotten item. For 360° turns, participants were encouraged to turn naturally, avoiding spinning or military-style turns. All assessments were conducted barefoot in a well-lit open space, with research staff spotting for safety.

### Walking and turning data processing

Walking and turning metrics were collected using Opal wireless inertial sensors and quantified using validated software (Mobility Lab, V2) (Opals by Clario - APDM Wearable Technologies, Portland, OR, USA)^23^. The primary walking metrics included cadence (steps/min), double support time as a percent of total gait cycle time (%GCT), gait speed (m/s), and stride length (m). These metrics were selected based on their sensitivity to gait impairments in PwMS and their frequent use in both clinical and research settings to capture key aspects of spatiotemporal gait performance^24,25^. Gait speed and stride length have shown strong discriminative power in prior MS studies, while cadence and double support time provide additional insight into rhythm and stability during walking^24,26–29^. The primary turning measures for the 360° and 180° turns included turn angle (degrees), turn duration (s), and peak turn velocity (degrees/s), which are widely used to quantify turning dynamics and have been shown to differentiate between impaired and unimpaired mobility performance^3,24–26,30^. Turn angle reflects how closely an individual matches the prescribed turn amount (e.g., a 360° turn), with greater deviations, particularly those exceeding the target, are often associated with greater mobility function, and angles closer to the target indicating a more cautious movement strategy. Turn duration captures the total time required to complete a turn, where longer durations indicate reduced turning performance. Peak turn velocity represents the highest angular velocity achieved during the turn, with higher values generally reflecting more optimal turning performance^5,31^.

### MRI acquisition

Resting-state image acquisition was completed with a 3T Siemens MAGNETOM Prisma^fit^ (Siemens Medical Solutions, USA, Inc., Malvern, PA) MRI scanner using a 32-channel head coil and parallel imaging. The anatomical scan consisted of a high-resolution T1-weighted MPRAGE image (GRAPPA acceleration factor 2, voxel-size = 0.8 × 0.8 × 0.8mm^3^, TR = 2400ms, TI = 1000ms, TE = 2.07ms, flip angle = 8°, FoV = 256 mm). The resting-state images were acquired with a fast echo-planar imaging sequence with BOLD contrast (TR = 460ms, TE = 27.20ms, flip angle = 44°, 56 slices, slice thickness = 3.00 mm, acceleration factor 8, number of measurements = 1,044), totaling 8 min of scan time. While in the scanner and prior to initiating the resting-state sequence, participants were instructed to remain awake, focus their gaze on a projected fixation cross, and keep a clear and relaxed mind.

### MRI preprocessing

Preprocessing was conducted using CONN Toolbox (version 22.a) and MATLAB (R2023a, The MathWorks Inc, Natick, MA, USA)^32^. Functional and anatomical data underwent a standard preprocessing pipeline, including realignment, slice timing correction, outlier detection, segmentation, MNI-space normalization, smoothing, and denoising^33^. Functional data were realigned using the SPM realign and unwarp procedure, where all scans were co-registered to a reference image using a least squares approach and a 6-parameter (rigid body) transformation and resampled using b-spline interpolation to correct for motion and magnetic susceptibility interactions^34,35^. Outlier scans were identified using ART if framewise displacement exceeded 0.5 mm or global BOLD signal changes exceeded three standard deviations^36,37^. A total of 38 scans were flagged as outliers out of 59,470 total volumes across all participants (< 0.064%), indicating high overall data quality. A reference BOLD image was then generated by averaging non-outlier scans. Functional and anatomical data were normalized to MNI space, segmented into grey matter, white matter, and CSF, and resampled to 2 mm isotropic voxels using SPM unified segmentation and normalization algorithm with the IXI-549 tissue probability map template^38–40^. Functional data were smoothed using an 8 mm full-width half maximum Gaussian kernel.

To evaluate potential group differences in motion, we compared mean head motion between groups and found no significant differences (healthy controls: 0.031 ± 0.008 mm; PwMS: 0.034 ± 0.008 mm; *p* = 0.26), suggesting comparable head motion and scan quality between groups.

Denoising followed a standard pipeline, regressing out potential confounds: white matter (5 CompCor components), CSF (5 CompCor components), motion parameters and derivatives (12 factors), outlier scans (< 3 factors), session effects and derivatives (2 factors), and linear trends (2 factors)^33,37,41^. A bandpass filter (0.008–0.1 Hz) was applied to the BOLD timeseries^42^. CompCor components were estimated by averaging BOLD signals within eroded white matter and CSF masks, extracting principal components orthogonal to motion parameters and outlier scans^43,44^.

### Multivariate pattern analysis (MVPA)

MVPA was used to identify rs-fMRI patterns associated with mobility measures and group differences. This data-driven approach applies singular value decomposition to seed-based correlations, producing a low-dimensional representation of voxel-to-voxel connectivity^18^. To reduce dimensionality, a two-step principal component analysis (PCA) was performed: (1) 64 subject-specific components were retained, and (2) five eigenpatterns were selected, maintaining a 10:1 subject-to-component ratio. Independent F-tests were conducted on the five eigenpatterns to identify significant connectivity variations between groups for the four mobility conditions (normal and fast 360° turns, normal 180° turns, and straight-ahead walking). Separate models were developed for each condition, controlling for age. Clusters meeting k ≥ 50 voxels and FDR-corrected *p* < 0.05 were retained as seeds for post-hoc seed-to-voxel analyses. For the seed-to-voxel analyses, each mobility condition was analyzed separately, with associated variables included as predictors (gait: cadence, double support time, gait speed, stride length; turning: turn angle, turn duration, peak velocity). Age was retained as a covariate, with voxel-level *p* < 0.001 and FDR-corrected cluster-level *p* < 0.05. The associated network membership for significant clusters was based on the 7-network Yeo et al. (2011) atlas^22^.

### Statistical analysis

Statistical analyses were conducted using JMP Pro 17 and the CONN Toolbox, with an alpha level of 0.05. Group differences in categorical demographic variables were evaluated using chi-square tests, and independent t-tests were used for continuous variables.

For all mobility outcomes, separate mixed effects models using REML estimation were conducted for each dependent variable across the three conditions: straight walking, 180° turning, and 360° in-place turning. For all straight walking and 360° in-place turning metrics, models included age and sex as fixed-effect covariates and participant ID as a random effect. For 180° turn metrics, the total number of turns completed during the 2MWT was included as a covariate due to significant group differences in turn count (Wilcoxon Two-Sample Test, *p* = 0.027; mean ± SD: healthy controls = 4.18 ± 0.67, PwMS = 3.65 ± 0.95). Turn metrics were calculated as the average of all completed turns per participant. To evaluate model assumptions, linearity, homoscedasticity, and normality of residuals were evaluated using residual plots and Q–Q plots.

To assess associations between resting-state functional connectivity (rs-FC) and mobility performance, z-scored connectivity values from significant MVPA-derived seed clusters were correlated with mobility metrics from post-hoc seed-to-voxel results. Correlations were conducted separately for PwMS and healthy controls. Pearson correlations were used for normally distributed data, and Spearman rank correlations were applied for non-normally distributed variables (assessed via Shapiro-Wilk tests and Q–Q plots). Correlation strength was classified for both types as follows: very strong (± 0.90–1.00), strong (± 0.70–0.89), moderate (± 0.50–0.69), weak (± 0.30–0.49), and negligible ( ± < 0.30)^45^. When appropriate, FDR correction was performed using the Benjamini-Hochberg procedure, with an adjusted p-value threshold of *p* < 0.05 for mixed-effect model results and seed-based associations.

## Results

### Participant characteristics

Table 1 summarizes participant characteristics. Groups did not differ significantly in demographics. All PwMS had an RRMS diagnosis, with an average disease duration of 11.93 years and an Expanded Disability Status Scale (EDSS) range of 0–5.5.

**Table 1:** Participant characteristics and demographics for each group.

Variable	Healthy control	Multiple sclerosis	*p*-value
Sex, female/male	20/8	20/9	
Age (years), mean ± SD	47.53 ± 15.93	47.50 ± 12.18	0.99
Height (cm.), mean ± SD	169.27 ± 7.80	166.37 ± 7.62	0.16
Mass (kg.), mean ± SD	73.05 ± 14.02	68.73 ± 9.07	0.17
BMI, mean ± SD	25.41 ± 4.08	24.93 ± 3.71	0.65
Disease Duration (years), mean ± SD	–	11.93 ± 10.47	
EDSS, median [min-max]	–	4 [0-5.5]	

BMI body mass index, EDSS expanded disability status scale.

### Walking and turning differences between groups

PwMS exhibited significantly slower gait speeds and reduced stride lengths compared to controls during the 2MWT (*F*(1,50) = 6.25, FDR-*p* = 0.047; (*F*(1,40) = 13.31, FDR-*p* = 0.002, respectively), though cadence (*F*(1,42) = 0.36, FDR-*p* = 0.828) and double support time (*F*(1,53) = 3.46, FDR-*p* = 0.206) did not differ between groups. No significant group differences were found for 180° turns during the 2MWT in turn duration (*F*(1,14.3) = 0.22, FDR-*p* = 0.648), peak turn velocity (*F*(1,22.1) = 0.005, FDR-*p* = 0.947) or turn angle (*F*(1,17.7) = 0.65, FDR-*p* = 0.619), suggesting similar turning strategies between groups. For fast-pace 360° turns, PwMS had longer turn durations (*F*(1,8) = 23.96, FDR-*p* = 0.002), slower peak velocities (*F*(1,53) = 24.50, FDR-*p* < 0.001), and reduced (i.e., nearer to 360°) turn angles (*F*(1,53) = 5.96, FDR-*p* = 0.018) compared to controls. For natural-pace 360˚ turns, PwMS also exhibited longer turn durations (*F*(1,43) = 5.75, FDR-*p* = 0.031), slower peak velocities (*F*(1,53) = 8.39, FDR-*p* = 0.008), and reduced turn angles (*F*(1,53) = 8.13, FDR-*p* = 0.009) compared to controls. Walking and turning results are summarized in Supplemental Table 1.

### Walking and functional connectivity pattern differences between groups

**Fig. 1:**
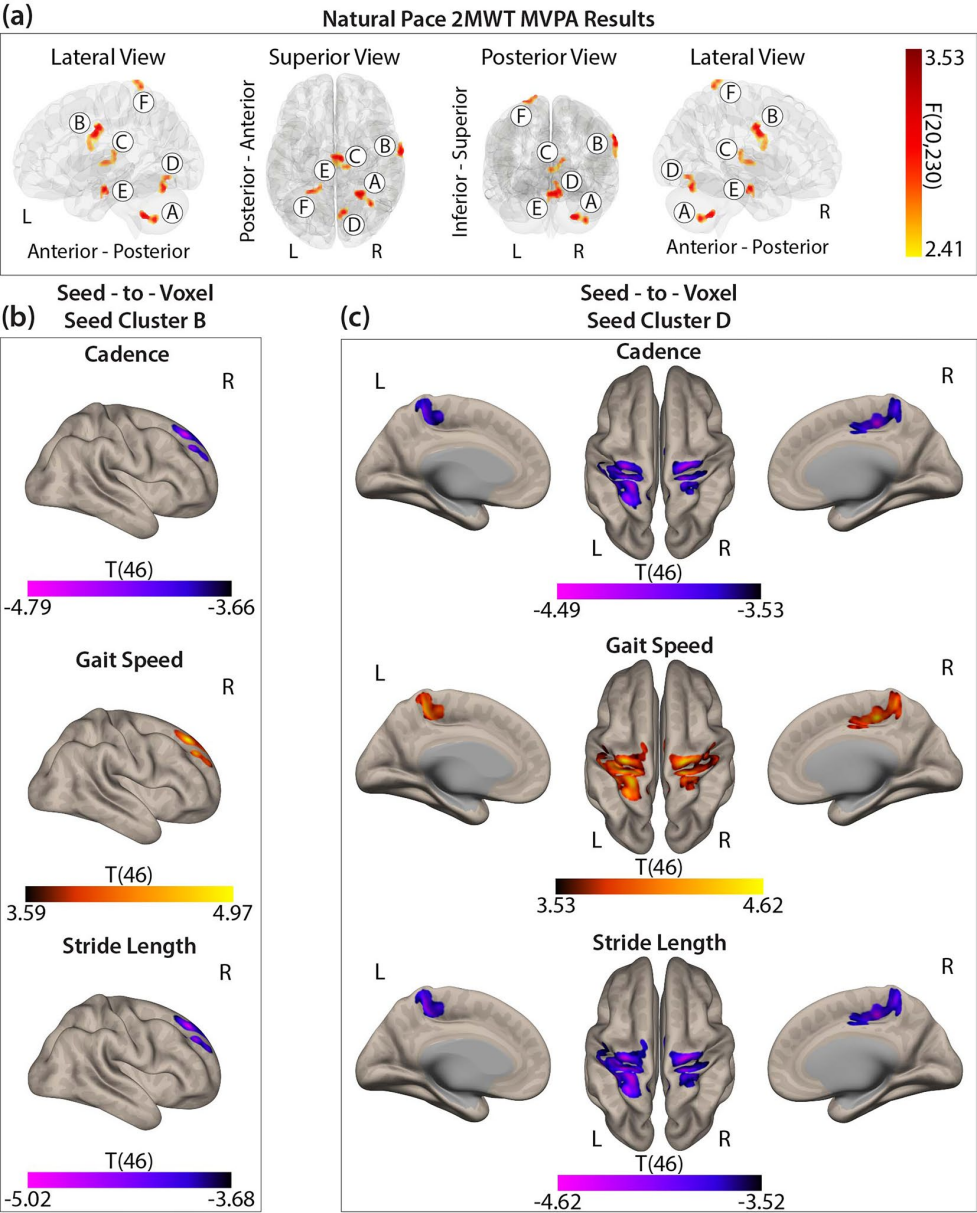
(**a**) Six seed clusters identified from the MVPA. (**b**) Post-hoc seed-to-voxel correlation clusters for cadence, gait speed, and stride length displaying differences between groups for seed cluster B. (**c**) Post-hoc seed-to-voxel correlation clusters for cadence, gait speed, and stride length differences between groups for seed cluster D. Color bar represents statistical significance.

Using MVPA, six distinct clusters (*F*(20,230) > 2.40, k ≥ 48) were identified, indicating functional connectivity patterns associated with group differences in linear walking variables (Supplemental Table 2). Seed cluster A was located in the right cerebellar hemisphere (cerebellum 2, 7, 8, 9) (peak MNI: +26, -48, -44) and was not associated with a particular Yeo et al. functional network. Seed cluster B was identified in the right pre- and post-central gyrus (peak MNI: +66, -08, + 36) within the primary motor and somatosensory cortex, linked to the somatomotor network (SN). Seed cluster C was located in the left and right thalamus (peak MNI: +12, -22, + 14), a location not associated with a particular Yeo et al. functional network. Seed cluster D was located in the right cerebellar hemisphere (cerebellum 6), the cerebellar vermis (vermis 6), and the right lingual gyrus (peak MNI: +08, -68, -18), linked with the visual network (VIS). Seed cluster E was located within the brainstem (peak MNI: +06, -14, -20) a location not linked to a Yeo et al. functional network. Seed cluster F did not meet the minimum size of k ≥ 50 and therefore will not be reported. Additionally, only seed clusters B and D maintained significance in the post-hoc seed-to-voxel analyses. See Fig. 1a for cluster visualization in MNI space.

#### Post-hoc seed cluster B: self-selected pace 2MWT

For seed cluster B (right pre- and postcentral gyrus; SN), cadence, gait speed, and stride length independently resulted in similar cluster formations spanning the DMN, FPN, and VAN. The results showed opposing correlation patterns between groups. For cadence and stride length the results showed anticorrelations in healthy controls and positive correlations in PwMS, while the opposite was observed for gait speed. Full region details are provided in Fig. 1b and Supplemental Table 2.

#### Post-hoc seed cluster D: self-selected pace 2MWT

For seed cluster D (right cerebellum, vermis, right lingual gyrus; VIS), cadence, gait speed, and stride length also independently resulted in similar cluster formations spanning the SN, VAN, and DAN. Again, cadence and stride length showed anticorrelations in healthy controls and positive correlations in PwMS while the opposite was observed for gait speed. See Fig. 1c and Supplemental Table 2 for full regional breakdowns.

### Turning and functional connectivity pattern differences between groups

#### 180˚ self-selected pace turning and functional connectivity

No significant seeds were associated with differences between groups for 180˚ turning measures (all seeds k < 50).

#### 360˚ self-selected natural pace turning and functional connectivity

**Fig. 2:**
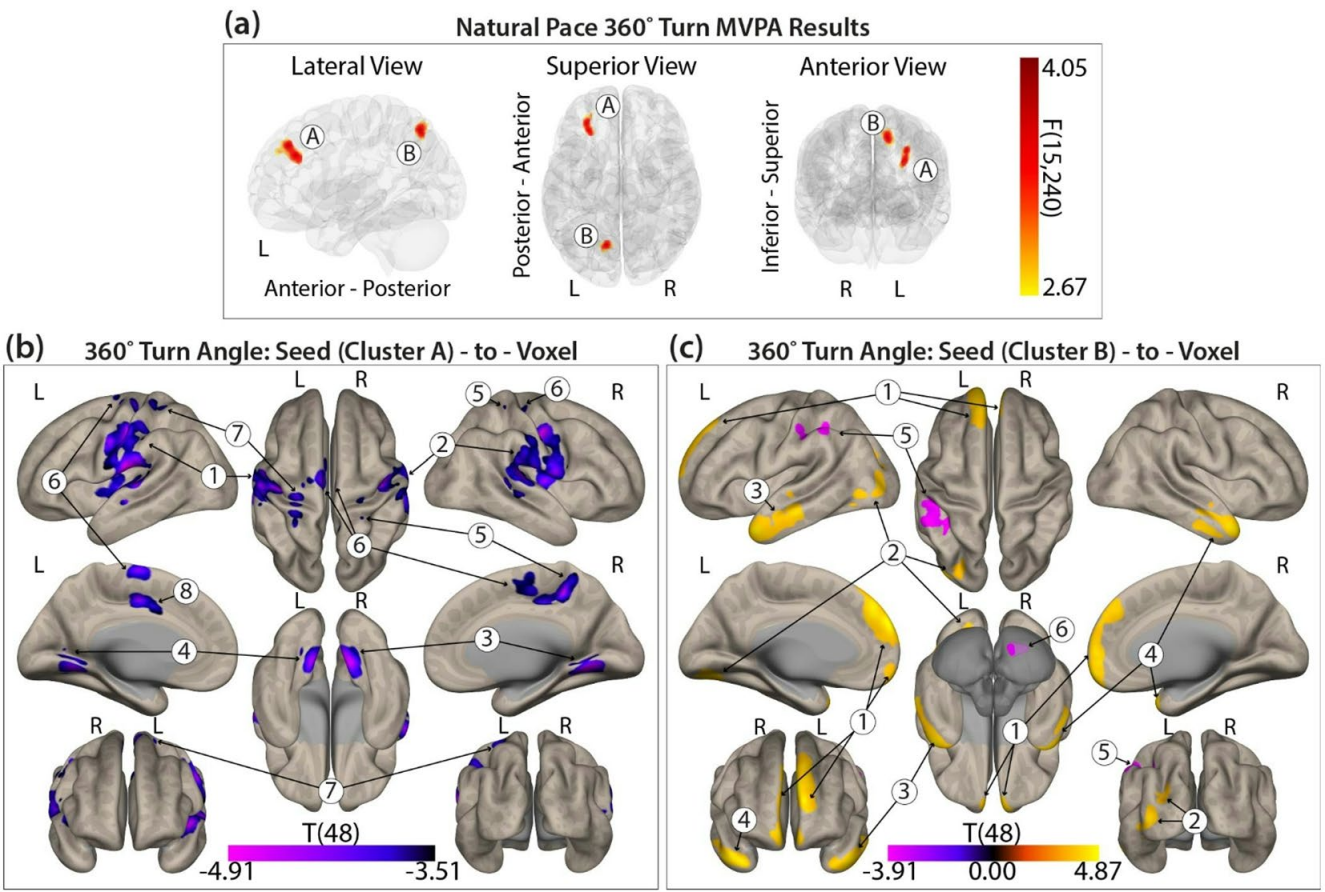
(**a**) Three seed clusters identified from the MVPA. (**b**) Post-hoc seed-to-voxel correlation clusters for 360° turn angle differences between groups for Seed Cluster (A) (**c**) Post-hoc seed-to-voxel correlation clusters for 360° turn angle differences between groups for Seed Cluster (B) Color bar represents statistical significance.

Two distinct clusters (*F*(15,240) > 2.65, k ≥ 99) were identified, showing functional connectivity patterns associated with self-selected natural pace 360˚ turning differences between groups (Fig. 2a, Supplemental Table 3). Seed cluster A was located in the left middle frontal gyrus and left frontal pole (peak MNI: -30, + 38, +40), overlapping with the VAN. Seed cluster B was found in the left superior lateral occipital cortex and precuneus (peak MNI: -12, -68, + 52), overlapping with the DAN. See Fig. 2a and Supplemental Table 3 for full regional breakdowns.

##### Post-hoc seed cluster a: self-selected natural pace turning

For seed cluster A (left middle frontal gyrus and frontal pole), turn angle was the only variable that retained significance. Eight clusters spanning the SN, VAN, VIS, and DMN showed opposing correlation patterns between groups, with anticorrelations in healthy controls and positive correlations in PwMS. Full region and network details are presented in Fig. 2b and Supplemental Table 3.

##### Post-hoc seed cluster B: self-selected natural pace turning

For seed cluster B (left superior lateral occipital cortex and precuneus), turn angle again showed opposing correlation patterns across six clusters. Healthy controls exhibited positive correlations in the DMN, VIS, and limbic network, while PwMS showed anticorrelations; the reverse pattern was seen in cerebellar and attentional networks. Details are provided in Fig. 2c and Supplemental Table 3.

**Fig. 3:**
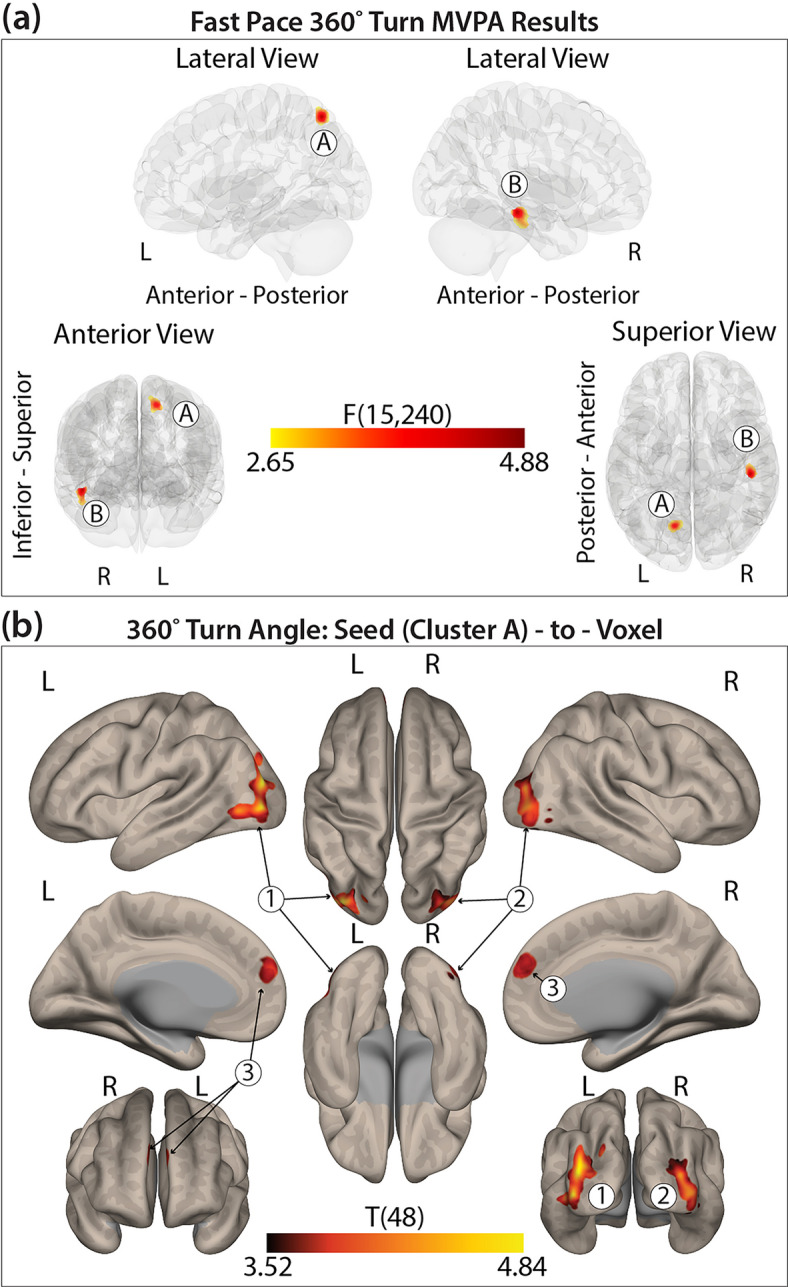
(**a**) Two seed clusters identified from the MVPA. (**b**) Post-hoc seed-to-voxel correlation clusters for 360° turn angle differences between groups for Seed Cluster A. Color bar represents statistical significance.

#### 360° self-selected fast pace turning and functional connectivity

Two distinct clusters (*F*(15,240) > 2.65, k ≥ 83) were identified, indicating functional connectivity patterns associated with self-selected fast pace 360° turning differences between groups. Seed cluster A was located in the left superior lateral occipital cortex and precuneus (peak MNI: − 14, − 68, + 56), overlapping with the dorsal attention network. Seed cluster B was found in the right inferior temporal gyrus, posterior region (peak MNI: + 46, − 26, − 14), which did not correspond with a defined network and did not maintain significance in the post-hoc analysis (Fig. 3a, Supplemental Table 4).

##### Post-hoc seed cluster A: fast pace turning

For seed cluster A (left superior lateral occipital cortex and precuneus; DAN), three clusters in the VIS and DMN showed group-specific correlation patterns: healthy controls had positive correlations, while PwMS showed anticorrelations. Seed Cluster B did not achieve significance following the post-hoc seed-to-voxel analysis. See Fig. 3b and Supplemental Table 4 for full region descriptions.

### Correlation results between gait variables and functional connectivity

We conducted post-hoc correlation analyses to examine whether functional connectivity strength in seed clusters B and D was associated with gait metrics that remained significant following the seed-to-voxel analysis (cadence, gait speed, and stride length). Despite consistent group-level connectivity differences observed across all three gait metrics, the within-group associations between functional connectivity and gait performance were weak or negligible in both PwMS and healthy controls (Supplemental Table 5). These results suggest that while gait-related network reorganization differentiates PwMS from controls, the magnitude of functional connectivity differences does not linearly reflect individual variability in gait performance within each group. This pattern may reflect group-level neural adaptations, for instance, compensatory or disease-related connectivity modifications that are not directly proportional to walking performance across participants.

### Correlation results between self-selected natural pace turning variables and functional connectivity

Correlation analyses for self-selected pace 360° turns revealed one significant correlation per seed cluster in PwMS, while healthy controls exhibited multiple significant associations across both seeds (Supplemental Table 6).

**Fig. 4:**
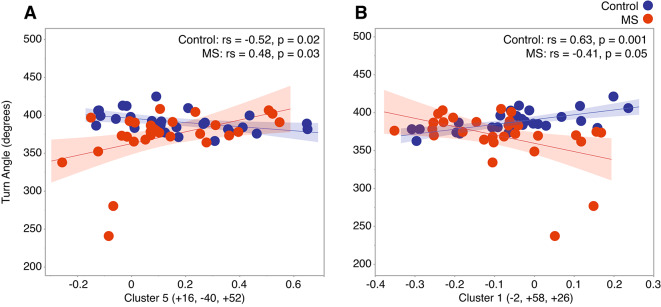
(**A**) In healthy controls, stronger anticorrelations within the VAN were associated with larger 360° turn angles. In PwMS, stronger positive correlations were linked to larger turn angles. (**B**) In healthy controls, stronger positive correlations between the DMN, limbic network, and the DAN were associated with larger 360° turn angles. In PwMS, stronger anticorrelations between these networks were linked to larger turn angles.

For seed cluster A (left middle frontal gyrus and left frontal pole; VAN), a significant association in both groups were observed within the VAN. In healthy controls, anticorrelated functional connectivity was associated with larger turn angles (> 360°) (rs(29) = − 0.52, *p* = 0.02), while in PwMS, positive connectivity within the VAN was linked to larger turn angles (> 360°) (rs(29) = 0.48, *p* = 0.03) (Fig. 4A).

For seed cluster B (left superior lateral occipital cortex and precuneus; DAN), positive connectivity with clusters spanning the DMN and limbic network was associated with larger turn angles in healthy controls (rs(29) = 0.63, *p* = 0.001). In contrast, in PwMS, greater positive functional connectivity within the DMN and limbic network was associated with smaller turn angles (rs(29) = − 0.41, *p* = 0.05) (Fig. 4B).

### Correlation results between self-selected fast pace turning variables and functional connectivity

**Fig. 5:**
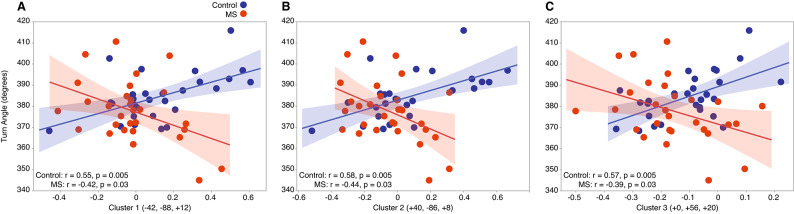
(**A**–**B**) In healthy controls, stronger positive connectivity between the VIS and the DAN was associated with larger 360° turn angles, while in PwMS, stronger anticorrelations in these regions were linked to larger turn angles. (**C**) In healthy controls, stronger positive connectivity between the DMN and the DAN was associated with larger 360° turn angles, whereas in PwMS, stronger anticorrelations in these regions were linked to larger turn angles.

For self-selected fast pace 360° turns, we identified significant but distinct connectivity patterns between groups (Supplemental Table 7). For seed cluster A (left superior lateral occipital cortex and precuneus; DAN), healthy controls exhibited positive connectivity between the DAN, VIS, and DMN, which was associated with larger turn angles (r(28) ≤ 0.58, *p* ≤ 0.005). Conversely, in PwMS, stronger anticorrelations between the DAN, VIS, and DMN were associated with smaller turn angles (r(29) ≤ − 0.44, *p* ≤ 0.03) (Fig. 5A-C).

## Discussion

### Walking and turning

This study demonstrated significant spatiotemporal differences in walking and turning between PwMS and healthy controls. PwMS exhibited slower gait speed and reduced stride length, though cadence and double support time did not significantly differ, aligning with prior research^28^.

No significant differences were found in 180° turn performance, diverging from studies reporting longer turn durations in PwMS during instrumented Timed Up and Go (TUG) tasks^3^. This discrepancy is likely task-related, as the TUG and 2MWT impose different task demands. In contrast, our 360° turn findings align with prior research, showing significantly longer turn durations in PwMS^46^.

While group differences in 360° turn velocity and angle remain inconsistently reported, our findings suggest that PwMS exhibit altered turning strategies specifically for in-place 360° turns, but not for 180° turns performed during walking trials. Notably, the reduced 360° turn angle in PwMS was accompanied by slower turn velocity and longer turn duration, suggesting that a smaller turn angle may serve as a marker of impaired turning performance. These results underscore the influence of task complexity and turn style on mobility impairments in PwMS.

### Mobility and functional connectivity differences between groups

PwMS consistently exhibit altered rs-FC across multiple brain networks, correlating with motor function, cognitive performance, and disease severity^7,14^. Prior research suggests functional connectivity changes worsen with disease progression, yet most studies focus on specific networks, such as the DMN or SN, rather than extending their analysis to investigate the whole brain^17,47–50^. This network-specific focus limits the understanding of how connectivity alterations across the brain influence mobility in PwMS. To address this gap, we applied a data-driven, whole-brain MVPA approach to investigate functional connectivity differences related to linear and non-linear walking metrics. This comprehensive analysis allowed us to identify novel connectivity patterns associated with mobility performance, advancing current knowledge of motor impairments in PwMS.

#### Gait and functional connectivity

Our findings revealed group-level differences in rs-FC patterns associated with cadence, gait speed, and stride length based on MVPA and seed-to-voxel analyses. These three gait metrics were linked to connectivity differences involving seeds in the SN (seed cluster B; right pre- and postcentral gyrus) and VIS (seed cluster D; right cerebellum, vermis, and lingual gyrus). For seed cluster B, all three gait variables independently showed similar connectivity patterns with regions spanning the DMN, FPN, and VAN, particularly within the right frontal pole and superior frontal gyrus. Similarly, seed cluster D revealed consistent group differences involving the SN, DAN, and VAN, with prominent clusters in the bilateral pre- and postcentral gyri.

However, contrary to our original hypothesis and prior findings, post-hoc correlation analyses did not identify significant associations between rs-FC within these clusters and individual gait metrics for either group. Indicating that while PwMS and controls exhibit distinct connectivity patterns in motor- and attention-related networks, such differences may not directly correspond to individual variability in gait performance during steady-state linear walking. Instead, these connectivity alterations may reflect broader group-level compensatory or disease-related reorganization rather than specific predictors of gait behavior within individuals.

Nonetheless, these findings expand on previous research showing increased somatomotor connectivity in PwMS, which has been linked to disease severity and motor dysfunction^7,51,52^. Additionally, while motor lateralization is well-known in healthy adults, PwMS might exhibit more pronounced or functionally different lateralized connectivity due to compensatory plasticity or structural damage^12,53–55^. Our results support this by showing lateralized differences between groups, especially in right hemisphere motor, cerebellar, and visual areas. Future research should examine whether these lateralized changes are adaptive or maladaptive in network function.

#### Turning and functional connectivity

In contrast to steady-state linear walking, turning performance, particularly turn angle, demonstrated significant associations with rs-FC in both PwMS and controls. Significant group-level connectivity patterns were identified for self-selected natural pace 360° turns in seed clusters located in the left middle frontal gyrus and frontal pole (VAN), and the left superior lateral occipital cortex and precuneus (DAN). Notably, the DAN seed was also implicated in fast-paced turning, suggesting that connectivity in this network is relevant to turning behavior regardless of speed.

For self-selected natural pace 360° turns, healthy controls showed anticorrelations between the VAN and SN, with stronger anticorrelations linked to larger turn angles (> 360°). In contrast, PwMS showed positive correlations in these networks, with stronger correlations associated with turn angles that more closely resembled those of controls. Previous research has also identified the VAN as a network vulnerable to disruption in PwMS, where increased connectivity has been connected to preserved cognitive performance, which may also relate to motor function^47,56^.

For seed cluster B (left superior lateral occipital cortex and precuneus; DAN), both groups showed associations with a target cluster spanning the DMN and limbic networks during natural pace 360° turning. Healthy controls exhibited positive connectivity associated with larger turn angles, whereas PwMS exhibited the opposite pattern, with stronger anticorrelations linked to larger turn angles. These findings may reflect reduced connectivity efficiency in PwMS, possibly due to disease-related white matter damage affecting network synchronization.

For fast-paced turning, both groups demonstrated associations between the DAN and regions within the VIS and DMN. Healthy controls continued to show positive correlations between connectivity and turn angle, while PwMS again exhibited anticorrelations, which were associated with smaller turn angles. Given the DAN’s role in top-down attentional control during goal-directed behaviors, the observed group differences may indicate reduced efficiency or impaired dynamic network engagement in PwMS under higher mobility demands. These findings align with prior studies reporting DAN dysfunction in PwMS and reinforce the potential of turning metrics to reveal meaningful differences in functional brain organization related to motor performance^47,57^.

When considered together, the findings from linear walking and turning tasks reveal a meaningful contrast in how rs-FC relates to mobility in PwMS and healthy controls. While group-level differences in rs-FC were observed for both gait and turning metrics, only the turning conditions yielded significant correlations between connectivity patterns and behavioral performance. Specifically, although cadence, gait speed, and stride length were associated with distinct rs-FC differences between groups these neural patterns did not show significant associations with individual variability in gait performance within either group. In contrast, turn angle during 360° in-place turning, showed robust and directionally distinct correlations with rs-FC patterns in both groups. PwMS consistently demonstrated anticorrelations or positive connectivity patterns that were directionally opposite to those observed in controls, particularly in the DAN, VAN, DMN, and SN. These findings suggest that turning may be more sensitive than linear walking to detecting functionally meaningful brain–behavior relationships, potentially due to its greater cognitive and postural demands. Turning, especially under dynamic or fast-paced conditions, may require more complex integration of attentional and sensorimotor networks. This contrast highlights the value of incorporating multifaceted mobility tasks, such as turning, in studies of brain–behavior relationships in neurological populations.

### Therapeutic implications

The current findings provide initial insights into the neural mechanisms underlying mobility impairments in PwMS, particularly highlighting how functional connectivity patterns differ between walking and turning tasks. While rs-FC differences between groups were observed for both gait and turning metrics, only 360° turning variables were meaningfully associated with connectivity patterns within the DAN, VAN, DMN, and SN. These results suggest that turning, which imposes greater cognitive and motor demands than steady-state walking, may be a more sensitive indicator of underlying neural dysfunction or compensation in PwMS.

Although the study was not designed to test a specific intervention, the observed associations highlight potential targets for future rehabilitation strategies. Interventions that enhance attentional control, sensorimotor integration, or large-scale network efficiency, for instance, task-specific balance and turning training, cognitive-motor dual-task paradigms, or non-invasive brain stimulation, may be especially relevant. Furthermore, the differential patterns of connectivity between groups underscore the importance of tailoring therapeutic approaches based on both specific mobility deficits and the corresponding neural substrates. Future longitudinal and interventional studies are needed to determine whether modifying these network-level features can enhance functional mobility outcomes in PwMS.

## Study limitations

Several limitations should be considered when interpreting these findings. First, our study included only individuals with RRMS with a median EDSS score of 4, limiting the generalizability of results to other MS phenotypes and disease severities. As such, caution is advised when extrapolating these findings to individuals with progressive MS or more severe disability levels. Moreover, the observed connectivity–mobility relationships may be influenced by the moderate disability status of our sample. It remains unclear whether individuals with milder or more advanced MS would demonstrate similar or divergent patterns of functional connectivity and mobility associations. Prior studies suggest that compensatory connectivity changes may occur early in the disease course, while later stages may be characterized by network inefficiency or disconnection^58^. Future studies should aim to include a broader spectrum of MS subtypes to determine whether these connectivity-mobility relationships persist across different disease stages.

Second, our analyses focused on identifying functional connectivity patterns associated with mobility measures rather than purely differentiating groups based on rs-FC alone. This hypothesis-driven approach prioritized mobility-related neural associations but may have overlooked alternative group-based connectivity differences unrelated to gait and turning performance. A complementary exploratory whole-brain analysis may reveal additional functional connectivity disruptions in PwMS that are not specifically linked to mobility. Lastly, while we aimed to reduce variability related to medication effects by screening all participants for medication use and excluding those with changes in medication within the three months prior to participation, we acknowledge that we did not explicitly model potential confounding variables such as medication type, fatigue levels, or cognitive function. These factors are known to influence both rs-FC and mobility performance and may have contributed to variability in our findings. Their exclusion was due to study design and statistical power limitations. Incorporating such variables in future studies would provide a more comprehensive understanding of the brain–behavior relationships in PwMS and help disentangle disease-specific neural signatures from modifiable or state-dependent influences. Therefore, future investigations should systematically assess and include these confounding variables to improve the specificity and interpretability of connectivity–mobility associations in MS.

## Conclusions

This study provides new insights into the neural correlates of mobility impairments in PwMS by demonstrating distinct associations between rs-FC and both linear walking and non-linear turning performance. Our findings highlight spatiotemporal gait differences, with PwMS exhibiting slower gait speeds and reduced stride lengths but no differences in cadence or double support time. Additionally, no differences in 180° turns were observed, whereas 360° turns revealed significant group differences, emphasizing the impact of task complexity on turning performance in PwMS. Importantly, our functional connectivity analyses identified differential associations between mobility performance and connectivity patterns within the SN, VAN, and DAN. The prefrontal, occipital, precentral, and postcentral cortices emerged as key regions implicated in mobility impairments. These findings suggest that compensatory mechanisms or disease-related reorganization may underlie altered functional connectivity in PwMS. Collectively, our results underscore the importance of a whole-brain approach to understanding the neural mechanisms underlying mobility dysfunction in PwMS.

## Supplementary Information

Below is the link to the electronic supplementary material.

Supplementary Material 1

## Data Availability

Anonymized data supporting the findings of this study can be made available to interested parties upon reasonable request, subject to conditions of research ethics and restrictions according to participant consent by contacting the corresponding author.
