# Supplementary material for: Resting-state functional connectivity correlates of gait and turning performance in multiple sclerosis: a multivariate pattern analysis
Source: Sci Rep. 2025 Oct 20;15:36500. doi: 10.1038/s41598-025-21102-6 (PMC12537983; doi:10.1038/s41598-025-21102-6)
Supplement: Supplementary file 1 — Supplementary Material 1 [file 41598_2025_21102_MOESM1_ESM.pdf]

Supplemental Table 1. Walking and Turning Differences Between Groups

Table 1. Between-group differences, adjusting for age and sex for each turn measure and variable. In addition to age and sex, number of turns was adjusted for, for 180˚ turn variables.

Turn Variables	Healthy Control	Multiple Sclerosis	FDRp-value
**2MWT Natural Pace**			
Cadence (steps/min), EMM ± SE	115.90 ± 1.88	114.13 ± 1.81	0.828
Double Support (%GCT), EMM ± SE	19.26 ± 0.65	20.85 ± 0.64	0.206
Gait Speed (m/s), EMM ± SE	1.23 ± 0.04	1.10 ± 0.03	**0.047**
Stride Length (m), EMM ± SE	1.28 ± 0.03	1.14 ± 0.03	**0.002**
**180˚ Natural-Paced Turns**			
Turn Duration (s), EMM ± SE	2.14 ± 0.06	2.18 ± 0.06	0.648
Turn Velocity (˚/s), EMM ± SE	202.75 ± 6.26	203.32 ± 6.24	0.947
Turn Angle (˚), EMM ± SE	184.24 ± 1.74	182.47 ± 1.73	0.619
**360˚ Fast-Paced Turn**			
Turn Duration (s), EMM ± SE	1.90 ± 0.1	2.57 ± 0.1	**0.002**
Turn Velocity (˚/s), EMM ± SE	355.82 ± 11.09	283.56 ± 10.85	**0.0001**
Turn Angle (˚), EMM ± SE	382.57 ± 2.08	375.87 ± 2.04	**0.018**
**360˚ Natural Pace Turn**			
Turn Duration (s), EMM ± SE	2.98 ± 0.13	3.45 ± 0.12	**0.031**
Turn Velocity (˚/s), EMM ± SE	223.69 ± 7.44	195.32 ± 7.28	**0.008**
Turn Angle (˚), EMM ± SE	386.14 ± 4.81	368.08 ± 4.71	**0.009**

Notes: two-minute walk test (2MWT); percent gait cycle time (%GCT); data presented as estimated marginal means (EMMs) ± standard error (SE).

Supplemental Table 2. Walking-related MVPA cluster seeds and post-hoc functional connectivity patterns (only k > 50)

Peak coordinates (MNI)	Cluster #	Gait Variable	Peak brain regions per cluster ≥ 50 voxels (k) (% of the cluster)	Associated Brain Network (Cluster (k) ≥ 50)								
				+ /- FC	VIS	SN	FPN	DAN	VAN	DMN	LIMBIC	k
+26 -48 -44	***A|E***		***R Cereb8 (83%) / R Cereb7 (10%) / R Cereb9 (2%) / R Cereb2 (1%) seed***									***100***
+66 -08 +36	***B|E***		***R PreCG (26%) / R PostCG (24%) seed***			***59***						***88***
(+24 +48 +44)	I|I	Cadence	R FP (84%) / R SFG (2%)	HC (-) / MS (+)			34		23	355		458
(+24 +48 +44)	I|I	Gait Speed	R FP (84%) / R SFG (2%)	HC (+) / MS (-)			35		30	378		490
(+24 +48 +44)	I|I	Stride Length	R FP (82%) / R SFG (3%)	HC (-) / MS (+)			46		35	399		529
+12 -22 +14	***C|E***		***R Thalamus (71%) / L Thalamus (29%) seed***									***87***
+08 -68 -18	***D|E***		***R Cereb6 (52%) / Ver6 (27%) / R LG (21%) seed***		***6***							***67***
(+12 -26 +50)	I|I	Cadence	L PostCG (36%) / L PreCG (11%) / R PostCG (12%) / R PreCG (14%)	HC (-) / MS (+)		1932		55	88			2924
(+12 -26 +50)	I|I	Gait Speed	L PostCG (32%) / L PreCG (13%) / R PostCG (15%) / R PreCG (15%)	HC (+) / MS (-)		2742		67	118			4085
(+12 -26 +50)	I|I	Stride Length	L PostCG (35%) / L PreCG (13%) / R PostCG (13%) / R PreCG (13%)	HC (-) / MS (+)		2522		74	123			3790
+06 -14 -20	***E|E***		***Brainstem (26%) seed***									***61***

Note: Seed clusters represented in bold text. Abbreviations: cerebellum (Cereb); lingual gyrus (LG); precentral gyrus (PreCG); postcentral gyrus (PostCG); frontal pole (FP); superior frontal gyrus (SFG); vermis (Ver).

Supplemental Table 3. Self-selected pace 360˚ turn-related MVPA cluster seeds and post-hoc functional connectivity patterns (only k > 50)

Peak coordinates (MNI)	Cluster #	Peak brain regions per cluster ≥ 50 voxels (k) (% of the cluster)	+ /- FC	Associated Brain Network (Cluster (k) ≥ 50)							
				VIS	SN	FPN	DAN	VAN	DMN	LIMBIC	k
- 30 + 38 + 40	***A|B***	***L MidFG (48%) / L FP (38%) seed***						***119***			***144***
(-48 -14 -02)	1|8	L PostCG (26%) / L CO (20%) / L PreCG (12%) / L PT (6%) / L PO (6%) / L HG (5%) / L PP (3%) / L IC (3%) / L WM (18%)	HC (-) / MS (+)		1897			291			2743
(+48 -32 +12)	2|8	R CO (19%) / R PreCG (18%) / R PO (14%) / R PT (13%) / R IC (6%) / R PostCG (5%) / R WM (22%)	HC (-) / MS (+)		1313			287			2037
(+18 -58 -06)	3|8	R LG (93%)	HC (-) / MS (+)	356							389
(-16 -60 -04)	4|8	L LG (79%)	HC (-) / MS (+)	274							323
(+16 -40 +52)	5|8	Precuneous (19%) / R PostCG (18%) / R WM (44%)	HC (-) / MS (+)					178			305
(-10 -08 +68)	6|8	R SMA (21%) / R WM (31%)	HC (-) / MS (+)		179						267
(-24 -24 +64)	7|8	L PreCG (52%) / L PostCG (48%)	HC (-) / MS (+)		154						229
(-10 -10 +46)	8|8	AC (43%) / L SMA (32%) / L WM (25%)	HC (-) / MS (+)		158						209
- 12 - 68 + 52	***B|B***	***L sLOC (68%) / Precuneous (18%) seed***					***82***				***99***
(-02 +58 +26)	1|6	L FP (35%) / R FP (21%) / L SFG (13%) / R SFG (7%) / L PaCiG (3%) / R WM (21%)	HC (+) / MS (-)						1266	113	1674
(-16 -86 -08)	2|6	L iLOC (23%) / L OFusG (22%) / L sLOC (11%) / L WM (39%)	HC (+) / MS (-)	412							813
(-40 +10 -36)	3|6	L TP (35%) / L pMTG (35%) / L aMTG (13%) / L WM (14%)	HC (+) / MS (-)						535	135	759
(+30 +20 -36)	4|6	R TP (61%) / R aMTG (10%) / R pMTG (9%) / R WM (19%)	HC (+) / MS (-)						264	226	587
(-54 -38 +46)	5|6	L aSMG (60%) / L WM (22%)	HC (-) / MS (+)			64	255				397
(+26 -72 -52)	6|6	R Cereb8 (62%) / R Cereb7 (18%) / R WM (20%)	HC (-) / MS (+)								277

Note: Seed clusters represented in bold text. Abbreviations: middle frontal gyrus (MidFG); frontal pole (FP); postcentral gyrus (PostCG); central opercular cortex (CO); precentral gyrus (PreCG); posterior temporal cortex (PT); parieto-occipital cortex (PO); Heschl's gyrus (HG); insular cortex (IC); white matter (WM); lingual gyrus (LG); supplementary motor area (SMA); anterior cingulate gyrus (AC); superior frontal gyrus (SFG); inferior lateral occipital cortex (iLOC); occipital fusiform gyrus (OFusG); superior lateral occipital cortex (sLOC); temporal pole (TP); posterior middle temporal gyrus (pMTG); anterior middle temporal gyrus (aMTG); anterior supramarginal gyrus (aSMG); cerebellum (Cereb).

Supplemental Table 4. Fast pace 360˚ turn-related MVPA cluster seeds and post-hoc functional connectivity patterns (only k > 50)

Peak coordinates (MNI)	Cluster #	Peak brain regions per cluster ≥ 50 voxels (k) (% of the cluster)	+ /- FC	Associated Brain Network (Cluster (k) ≥ 50)							
				VIS	SN	FPN	DAN	VAN	DMN	LIMBIC	k
- 14 - 68 + 56	***A|A***	**L sLOC (92%) / Precuneous (6%) seed**					**78**				**88**
(-42 -88 +12)	1|3	L iLOC (41%) / L sLOC (33%) / L OP (11%) / L WM (15%)	HC (+) / MS (-)	767							990
(+40 -86 +08)	2|3	R iLOC (56%) / R sLOC (22%) / R WM (19%)	HC (+) / MS (-)	582							776
(+00 +56 +20)	3|3	R SFG (27%)	HC (+) / MS (-)						173		176

Note: Seed cluster represented in bold text. Abbreviations: superior lateral occipital cortex (sLOC); inferior lateral occipital cortex (iLOC); occipital pole (OP); superior frontal gyrus (SFG); white matter (WM)

Supplemental Table 5. Correlations between functional connectivity and gait variables

Peak coordinates (MNI)	Cluster #	Gait Variable	Peak brain region (% of the cluster)	Associated Network(s) (Clusters (k) > 50)	Control		MS	
					Spearman / Pearson	*p-FDR*	Spearman / Pearson	*p-FDR*
+26 -48 -44	***A|E***		***R Cereb8 (83%) / R Cereb7 (10%) / R Cereb9 (2%) / R Cereb2 (1%) seed***	***Not Associated***				
+66 -08 +36	***B|E***		***R PreCG (26%) / R PostCG (24%) seed***	***SN***				
(+24 +48 +44)	1|1	Cadence	R FP (84%) / R SFG (2%)	DMN; FPN; VAN	0.22	0.27	-0.03	0.90
(+24 +48 +44)	1|1	Gait Speed	R FP (84%) / R SFG (2%)	DMN; FPN; VAN	-0.005	0.98	0.20	0.31
(+24 +48 +44)	1|1	Stride Length	R FP (82%) / R SFG (3%)	DMN; FPN; VAN	-0.25	0.20	0.29	0.13
+12 -22 +14	***C|E***		***R Thalamus (71%) / L Thalamus (29%) seed***	***Not Associated***				
+08 -68 -18	***D|E***		***R Cereb6 (52%) / Ver6 (27%) / R LG (21%) seed***	***VIS***				
(+12 -26 +50)	1|1	Cadence	L PostCG (36%) / L PreCG (11%) / R PostCG (12%) / R PreCG (14%)	SN; VAN; DAN	0.03	0.87	0.06	0.76
(+12 -26 +50)	1|1	Gait Speed	L PostCG (32%) / L PreCG (13%) / R PostCG (15%) / R PreCG (15%)	SN; VAN; DAN	0.11	0.57	0.17	0.38
(+12 -26 +50)	1|1	Stride Length	L PostCG (35%) / L PreCG (13%) / R PostCG (13%) / R PreCG (13%)	SN; VAN; DAN	-0.34	0.86	0.24	0.21
+06 -14 -20	***E|E***		***Brainstem (26%) seed***	***Not Associated***				

Note: Seed clusters represented in bold text. cerebellum (Cereb); lingual gyrus (LG); precentral gyrus (PreCG); postcentral gyrus (PostCG); frontal pole (FP); superior frontal gyrus (SFG); vermis (Ver).

Supplemental Table 6. Correlations between functional connectivity and self-selected pace 360˚ turn angle

Peak coordinates (MNI)	Cluster #	Peak brain region (% of the cluster)	Associated Network(s) (Clusters (k) > 50)	Control		MS	
				Spearman	*p-FDR*	Spearman	*p-FDR*
-30, +38, +40	***A|B***	***L MidFG (48%) / L FP (38%) seed***	***VAN***				
(-48, -14, -02)	1|8	L PostCG (26%) / L CO (20%) / L PreCG / L WM (18%) / (12%) / L PT (6%) / L PO (6%) / L HG (5%)/ L PP (3%) / L IC (3%)	SN; VAN	**-0.59**	**0.01**	0.34	0.08
(+48, -32, +12)	2|8	R WM (22%) / R CO (19%) / R PreCG (18%) / R PO (14%) / R PT (13%) / R IC (6%) / R PostCG (5%)	SN; VAN	**-0.58**	**0.01**	0.34	0.08
(+18, -58, -06)	3|8	R LG (93%)	VIS	-0.36	0.08	0.37	0.08
(-16, -60, -04)	4|8	L LG (79%)	VIS	-0.38	0.08	0.06	0.77
(+16, -40, +52)	5|8	R WM (55%) / Precuneous (19%) / R PostCG (18%)	VAN	**-0.52**	**0.02**	**0.48**	**0.03**
(-10, -08, +68)	6|8	R SMA (21%)	SN	-0.4	0.07	0.32	0.09
(-24, -24, +64)	7|8	L PreCG (52%) / L PostCG (48%)	SN	**-0.56**	**0.01**	0.34	0.08
(-10, -10, +46)	8|8	AC (43%) / L SMA (32%) / L WM (25%)	SN	**-0.45**	**0.04**	0.41	0.06
-12, -68, +52	***B|B***	***L sLOC (68%) / Precuneous (18%) seed***	***DAN***				
(-02, +58, +26)	1|6	L FP (35%) / R FP (21%) / R WM (21%) / L SFG (13%) / R SFG (7%)	DMN; LIMBIC	**0.63**	**0.001**	**-0.41**	**0.05**
(-16, -86, -08)	2|6	L WM (39%) / L iLOC (23%) / L OFusG (22%) / L sLOC (11%)	VIS	**0.66**	**0.001**	-0.1	0.63
(-40, +10, -36)	3|6	L TP (35%) / L pMTG (35%) / L WM (14%) / L aMTG (13%)	DMN; LIMBIC	**0.65**	**0.001**	-0.09	0.63
(+30, +20, -36)	4|6	R TP (61%) / R WM (19%) / R aMTG (10%) / R pMTG (9%)	DMN; LIMBIC	**0.64**	**0.001**	-0.24	0.27
(-54, -38, +46)	5|6	L aSMG (60%) / L WM (22%)	DAN; FPN	**-0.56**	**0.004**	0.2	0.35
(+26, -72, -52)	6|6	R Cerebellum 8 (62%) / R WM (20%) / R Cerebellum 7 (18%)	Cerebellum	**-0.51**	**0.01**	0.27	0.24

Note: Seed clusters represented in bold text. Abbreviations: middle frontal gyrus (MidFG); frontal pole (FP); postcentral gyrus (PostCG); central opercular cortex (CO); precentral gyrus (PreCG); posterior temporal cortex (PT); parieto-occipital cortex (PO); Heschl's gyrus (HG); insular cortex (IC); white matter (WM); lingual gyrus (LG); supplementary motor area (SMA); anterior cingulate gyrus (AC); superior frontal gyrus (SFG); inferior lateral occipital cortex (iLOC); occipital fusiform gyrus (OFusG); superior lateral occipital cortex (sLOC); temporal pole (TP); posterior middle temporal gyrus (pMTG); anterior middle temporal gyrus (aMTG); anterior supramarginal gyrus (aSMG); cerebellum (Cereb).

Supplemental Table 7. Correlations between functional connectivity and fast pace 360˚ turn angle

Peak coordinates (MNI)	Cluster #	Peak brain region (% of the cluster)	Associated Network(s) (Clusters (k) > 50)	Control		MS	
				Pearson	*p*-FDR	Pearson	*p*-FDR

-14, -68, +56	*A|A*	*L sLOC (92%) / Precuneous (6%) seed*	*DAN*				
(-42, -88, +12)	1|3	L iLOC (41%) / L sLOC (33%) / L WM (15%) / L OP (11%)	VIS	**0.55**	**0.0048**	**-0.42**	**0.027**
(+40, -86, +08)	2|3	R iLOC (56%) / R sLOC (22%) / R WM (19%)	VIS	**0.58**	**0.0048**	**-0.44**	**0.026**
(+00, +56, +20)	3|3	R SFG (27%)	DMN	**0.57**	**0.0052**	**-0.39**	**0.034**

Note: Seed cluster represented in bold text. Abbreviations: superior lateral occipital cortex (sLOC); inferior lateral occipital cortex (iLOC); occipital pole (OP); superior frontal gyrus (SFG); white matter (WM)
